# Reduction of Coil-Crack Angle Sensitivity Effect Using a Novel Flux Feature of ACFM Technique

**DOI:** 10.3390/s22010201

**Published:** 2021-12-28

**Authors:** Ruochen Huang, Mingyang Lu, Ziqi Chen, Wuliang Yin

**Affiliations:** School of Electrical and Electronic Engineering, University of Manchester, Manchester M13 9PL, UK; ruochen.huang@manchester.ac.uk (R.H.); mingyang.lu@manchester.ac.uk (M.L.); wuliang.yin@manchester.ac.uk (W.Y.)

**Keywords:** alternating current field measurement (ACFM) testing, magnetic induction, finite element method (FEM) acceleration, coil-crack angle sensitivity, crack detection, conductive plate

## Abstract

Alternating current field measurement (ACFM) testing is one of the promising techniques in the field of non-destructive testing with advantages of the non-contact capability and the reduction of lift-off effects. In this paper, a novel crack detection approach was proposed to reduce the effect of the angled crack (cack orientation) by using rotated ACFM techniques. The sensor probe is composed of an excitation coil and two receiving coils. Two receiving coils are orthogonally placed in the center of the excitation coil where the magnetic field is measured. It was found that the change of the x component and the peak value of the z component of the magnetic field when the sensor probe rotates around a crack followed a sine wave shape. A customized accelerated finite element method solver programmed in MATLAB was adopted to simulate the performance of the designed sensor probe which could significantly improve the computation efficiency due to the small crack perturbation. The experiments were also carried out to validate the simulations. It was found that the ratio between the z and x components of the magnetic field remained stable under various rotation angles. It showed the potential to estimate the depth of the crack from the ratio detected by combining the magnetic fields from both receiving coils (i.e., the x and z components of the magnetic field) using the rotated ACFM technique.

## 1. Introduction

Surface crack detection is one of the most essential issues for researchers and engineers to improve the service life of equipment. A small crack can lead to an unreliable structure which can greatly shorten the service life of equipment. The techniques developed for the detection of the crack can effectively prevent unnecessary loss and damage, for example, the eddy current (EC) inspection [1,2,3,4] and the alternating current field measurement (ACFM) technique [5,6,7].

To quantitatively identify the position and size of surface cracks, various EC examination excitation strategies have been presented [1,2,3]. The ACFM technology was first employed in the oil and gas industry to detect surface fractures in previous decades [8,9]. It can inspect crack geometries (such as depth and length) with high precision and eliminate lift-off effects [10,11,12,13]. It also allows the sensor probe to identify surface defects in metals without having to remove the coatings. As a result of this feature, it can be successfully used in the inspection of rail axles, saving time and money. Both approaches inject alternating current into the excitation coils, causing an induced field in the examined material, while the receiving coils receive the signals from the induced field, similar to EC inspection. The induced eddy current is disturbed by the existence of the surface fracture, allowing the crack to be predicted by the received signal. The impedance of the probe due to the existence of the crack is measured in the EC inspection, whereas the (perturbance of) magnetic field is detected directly by the sensor probe in the ACFM [14,15]. To model the response of the magnetic field to crack perturbance, a customized finite element approach is used. To improve computing performance, methods such as the optimized initial preconditioner [16,17] and perturbed matrixed inversion [18] have been developed.

The ACFM approach for detecting surface cracks has been developed over the years in order to increase the reliability and sensitivity of the inspection. Inspection has been designed using a sensor probe array [19,20]. A single/multi-layered linear pick-up coil with its electronics, as it is attached below the inducer which generates the high-frequency interrogating field, offers high detection sensitivity (0.5–4 mm deep notches) in the inspection of surface cracks for the large metal plates. Theoretically, it can be made in any length; however, the resistance of the circuits limits the length of the sensor [19]. Li et al. presented a feed-through ACFM probe structure with an equal-spaced detecting sensor array that can detect the axial crack quantitatively and successfully scan the whole circumference of the pipe string [20]. Denoising the received signal from the measurements is also necessary in order to obtain better crack information outcomes. A magnetic core was appended to the driver coil and the proper wavelet function was chosen to execute the process of denoise. The signal characteristics were clearer after denoising by using the wavelet function [21].

Moreover, inverse problems for estimating the geometry information of the surface crack in the workpiece have been used in the ACFM techniques. Ravan et al. presented the method based on the artificial neural network scheme which can be used to predict the depth of surface cracks with random geometry and known length and direction of crack. However, the accuracy of depth prediction depends on the level of noise from the measurement data [22]. Fuzzy rules are also popularly applied in the identification of the crack [12,23,24]. Noroozi et al. utilized the fuzzy alignment algorithm (FAA) to effectively map the depth of the crack to the signal output from the probe. The FAA is capable of diminishing the impact aroused by the irrelevant training data and converging efficiently by setting a degree of freedom to manipulate the influence from the dataset. This method shows a good performance for the crack with arbitrary crack shape [12]. These methods require a certain amount of computation, and it is common to obtain the direction of the crack using the 2D scanning technique in advance.

During the measurement process, the vibration of the experiment setup would affect the accuracy of the measurement, leading to unnecessary damage. Gu et al. proposed a structural optimization method, and an absorber was used to reduce the vertical vibration of the machine [25]. Without knowing the crack orientation, the angle between the crack and the sensor probe would influence quantifying the crack dimension. In this paper, to analyze and reduce the effect of the angled coil (compared to the crack orientation) above the surface crack on conductive metals, a crack detection method by utilizing the rotated ACFM technique was proposed. For the proposed method, the designed sensor probe scanned above the sample plate in different angles. By using this method, the angle between the crack and the sensor probe could be eliminated. Simulations were conducted in the customized accelerated finite-element solver programmed in MATLAB and experiments were carried out to verify the proposed features.

## 2. Relationship between the Measured Magnetic Field and the Rotation Angle

### 2.1. Magnetic Field Calculated by the Accelerated Finite Element Analysis

As a versatile computation technique of simulating electromagnetic problems, the finite element method (FEM) is widely applied in diverse industrial applications in non-destructive testing. With the support of A-V edge-element formulation, FEM simulation was set up in MATLAB and by using the generated sample model, the magnetic field could be computed. In the formulation, the Galerkin method was employed to compute the vector potential (A) and scalar potential (*V*) of the whole domain [16,17,18]. The vector and scalar potentials in individual element satisfy:

(1) $$\begin{array}{c} \int_{\Omega_{c}}\nabla \times N_{i}\cdot v\nabla \times A^{n}d\Omega +\int_{\Omega_{c}}j\omega \sigma N_{i}\cdot A^{n}d\Omega +\int_{\Omega_{c}}j\omega \sigma N_{i}\cdot \nabla V^{n}d\Omega \\ =\int_{\Omega_{c}}\nabla \times N_{i}\cdot v_{0}\nabla \times A_{s}\begin{array}{cc} d\Omega & i \end{array}=1,2,\ldots ,6 \end{array}$$

(2) $$\int_{\Omega_{c}}j\omega \sigma \nabla L_{i}\cdot A^{n}d\Omega +\int_{\Omega_{c}}j\omega \sigma \nabla L_{i}\cdot \nabla V^{n}d\Omega =0i=1,2,\ldots ,4$$

where: $N_{i}$ is the edge interpolation function in *i*th edge; $L_{i}$ is the nodal interpolation function in *i*th edge; $\Omega_{c}$ is the metallic domain in the sample model; v is the sample reluctivity; σ is the sample conductivity; and $v_{0}$ denotes the reluctivity in air.

For each tetrahedral element, the interpolation functions are unique so that transformation of the coordinates is used to transform the global coordinates ($\lambda_{v},\;\lambda_{s}$) to the local coordinates (λ^v, λ^s). As a consequence, the interpolation functions can be given [18]:

(3) $$J=\left[\begin{array}{ccc} \frac{\partial x}{\partial \xi } & \frac{\partial y}{\partial \xi } & \frac{\partial z}{\partial \xi }\\ \frac{\partial x}{\partial \eta } & \frac{\partial y}{\partial \eta } & \frac{\partial z}{\partial \eta }\\ \frac{\partial x}{\partial \zeta } & \frac{\partial y}{\partial \zeta } & \frac{\partial z}{\partial \zeta } \end{array}\right]$$

(4) λv=J−1λ^v

(5) λs=J−1λ^s

(6) ∇×λv=1|J|JT∇×λ^v

where J is the Jacobian matrix, $\lambda_{v}$ is the vector component in the global coordinates, $\lambda_{s}$ is the scalar component in the global coordinates, λ^v is the vector component in the local coordinates, and λ^s is the scalar component in the local coordinates.

Therefore, employing Equations (1) and (2), a linear algebraic system equation can be expressed by using the stiffness matrix Q.

(7) $$Q=\left[\begin{array}{cc} K^{p\times p} & L^{p\times q}\\ M^{q\times p} & N^{q\times q} \end{array}\right]$$

(8) $$Q\left[\begin{array}{c} \left[\begin{array}{c} A_{1}\\ \vdots \\ A_{p} \end{array}\right]\\ \left[\begin{array}{c} V_{1}\\ \vdots \\ V_{q} \end{array}\right] \end{array}\right]=X$$

Here, p is the edge number, q is the vertex node number. K, which is associated with the summation of the first two terms of Equation (1), mainly dominates by the vector field, and contributes to the generation of the vector potential. L is the third term of Equation (2), controlling the flow of the eddy current as it encounters with the notch. M and N are the terms of the left-hand side of Equation (2), satisfying the conditions of magnetostatic field. X is the terms of the right-hand side of Equations (1) and (2), providing the background field of the entire system.

In order to hasten the computation speed, the accelerated method based on the property that the crack only disturbs its surrounding field was adopted [26]. In this method, the stiffness matrix Q can be rearranged and divided into four parts, $Q_{1}$, $Q_{2}$, $Q_{3}$, and $Q_{4}$. Here $Q_{1}$ is the matrix unaffected by the small perturbation, while $Q_{2}$, $Q_{3}$, and $Q_{4}$ are the matrices affected by the small perturbation. $S_{u}$ and $S_{c}$ are the field solution of the unaffected domain and affected domain respectively. $X_{u}$ and $X_{c}$ are the background field of the unaffected domain and affected domain. The system matrix equation turns to:

(9) $$\left[\begin{array}{cc} Q_{1} & Q_{2}\\ Q_{3} & Q_{4} \end{array}\right]\left[\begin{array}{c} S_{u}\\ S_{c} \end{array}\right]=\left[\begin{array}{cc} \left[\begin{array}{cc} K_{1} & L_{1}\\ M_{1} & N_{1} \end{array}\right] & \left[\begin{array}{cc} K_{2} & L_{2}\\ M_{2} & N_{2} \end{array}\right]\\ \left[\begin{array}{cc} K_{3} & L_{3}\\ M_{3} & N_{3} \end{array}\right] & \left[\begin{array}{cc} K_{4} & L_{4}\\ M_{4} & N_{4} \end{array}\right] \end{array}\right]\left[\begin{array}{c} \left[\begin{array}{c} A_{u}\\ V_{u} \end{array}\right]\\ \left[\begin{array}{c} A_{c}\\ V_{c} \end{array}\right] \end{array}\right]=\left[\begin{array}{c} X_{u}\\ X_{c} \end{array}\right]$$

Then due to the perturbation of the small crack, crack matrices are introduced, and the system is equal to:

(10) $$\left[\begin{array}{cc} Q_{1} & Q_{2}+∆Q_{2}\\ Q_{3}+∆Q_{3} & Q_{4}+∆Q_{4} \end{array}\right]\left[\begin{array}{c} S_{u}{}^{’}\\ S_{c}{}^{’} \end{array}\right]=\left[\begin{array}{c} X_{u}\\ X_{c} \end{array}\right]$$

where $∆Q_{12}$, $∆Q_{21},$ and $∆Q_{22}$ are the matrices influenced by the small perturbance, $S_{u}{}^{’}$ and $S_{c}{}^{’}$ are the solutions for the crack disturbed system equations.

By utilizing the fact that the perturbed field due to the crack can be localized to its surrounding area, the solutions can be obtained by Equation (11) which can effectively hasten the computation speed [26].

(11) $$\{\begin{array}{c} S_{u}{}^{’}=S_{u}\\ \left(Q_{4}+∆Q_{4}\right)S_{c}{}^{’}=X_{c}-\left(Q_{3}+∆Q_{3}\right)S_{u} \end{array}$$

Therefore, after obtaining the magnetic vector potential field $A_{p}$ along all the edges and electric scalar potential field $V_{q}$ on all the vertex of the entire crack system, the eddy current produced in the tested sample was equal as:

(12) $$J_{s}=\sigma E=-j\omega \sigma A-\sigma \nabla V\;$$

where E is the electric field contributed by both the vector and scalar potential field.

Based on the Biot–Savart law, the magnetic B field can be derived as:

(13) $$B=\frac{\mu_{0}}{4\pi }\int^{\;}\frac{J_{s}\times r^{\prime }}{{\left|r^{\prime }\right|}^{3}}dV$$

Here $\mu_{0}$ denotes the permeability in the vacuum, $J_{s}$ denotes the generated eddy current in the tested sample, and $r^{\prime }$ denotes the displacement vector from the current element to the computed point. Consequently, $B_{x}$ and $B_{z}$ can be described by the first and third components of the calculated magnetic B field and the ratio between $B_{z}$ and $B_{x}$ can be calculated.

### 2.2. Simulation Models

**Figure 1:**
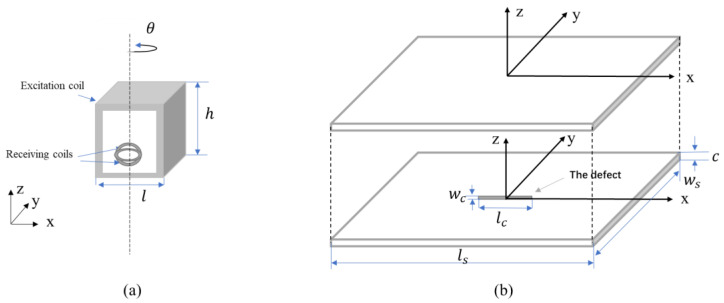
Simulation models (**a**) the rotation direction of the sensor probe (**b**) the stainless-steel plate with/without the crack.

Figure 1 shows the rotation direction of the sensor probe (rotates counterclockwise) and it scans above the non-magnetic tested sample with/without the crack. As shown in Table 1, the height and the length of the excitation coil were set to 3 and 4 mm, respectively. The radius of the receiving coils was 0.5 mm. The probe was placed 0.5 mm above the sample model. The conductivity of the sample plate was 1.4 MS/m with a length of 75 mm and a width of 40 mm. The thickness of the sample in the model was 2 mm and the length and width of the crack in the center of the plate were 10 and 0.25 mm, respectively.

### 2.3. Eddy Currents around Cracks Using the FEM Solver

**Figure 2:**
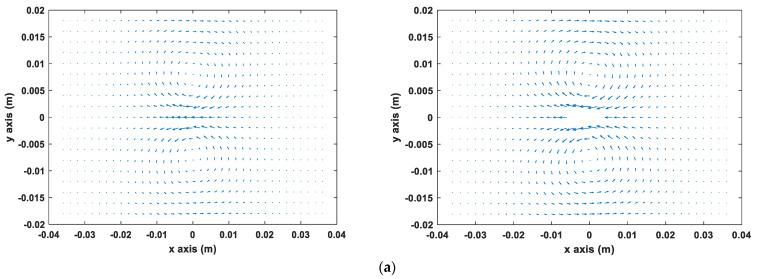

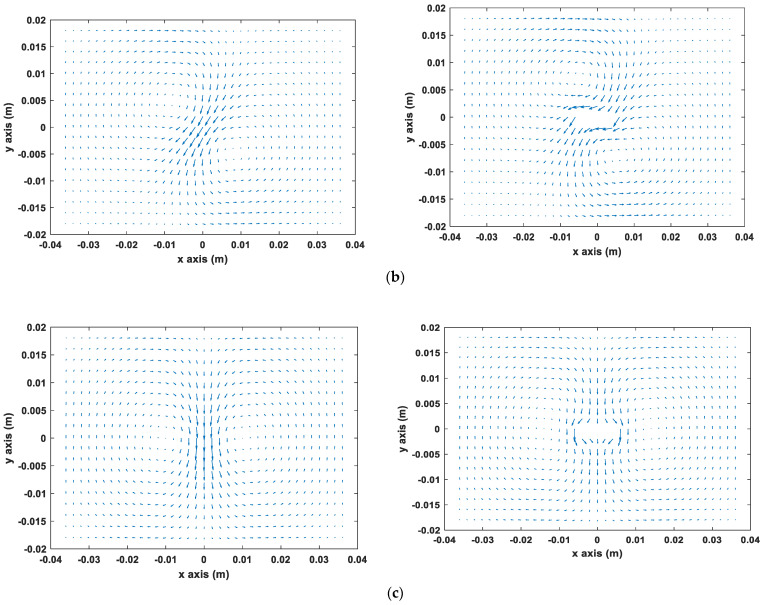
The flow of eddy current induced on the non-magnetic tested sample without/with the crack (**a**) rotation angle 0°, (**b**) rotation angle 45°, (**c**) rotation angle 90°.

The behavior of the eddy current around the crack on the sample plate was simulated using the accelerated FEM solver. The sensor probe was situated above the center of the sample plate with different rotation angles and the lift-off of 0.5 mm. Figure 2 shows the vector diagram of the eddy current flow under three rotation angles, 0°, 45°, and 90°, respectively.

As shown in Figure 2, it can be noted that the eddy current was distributed uniformly in the center of the sample plate and had the feature of symmetry. The eddy current flowed uniformly and continuously in the sample plate without the disturbance of the crack. When the eddy current encounters with a crack, it will flow around the edge of the crack. As can be seen in Figure 2a, when the sensor probe was parallel to X-axis (rotation angle 0°), the eddy current was hardly affected, then the probe rotated to 45°, the eddy current was perturbed and flowed according to the geometry of the crack. The strongest perturbation occurs when it rotated to 90°, as shown in Figure 2c. Consequently, the impact on the eddy current due to the crack for the sensor probe perpendicular to X-axis (rotation angle 90°) was strongest compared with other rotation angles, resulting in the significant change of the magnetic field. Due to the rotation of the sensor probe, the strength of the detected magnetic field was affected by the induced eddy currents varying. Therefore, it can be deduced that the weakest appeared at the angle of 0°/180° and the strongest appeared at the angle of 90°.

### 2.4. Coil Angle-Immune Feature on Crack Detection Using the Rotary Sensor Probe

**Figure 3:**
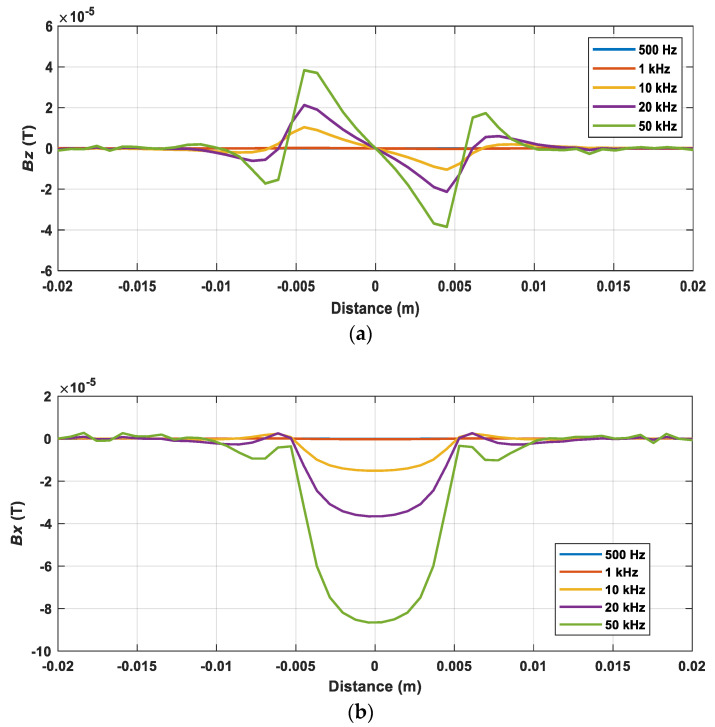
The magnetic B field received by the sensing probe under different excitation frequencies (**a**) $B_{z}$, (**b**) $B_{x}$.

To simulate the magnetic field due to the presence of the crack in the sample model, the sensor probe scanned across the crack along X-axis from (−20, 0, 0.5) to (20, 0, 0.5) mm (i.e., perpendicularly to the crack-rotation angle 90°). The model parameters listed in Table 1 were kept the same in the process of the entire simulation. Considering the effect of different depths, the crack in the center was evenly divided into 10 layers, from 0.2 to 2 mm. Figure 3 shows the received magnetic B field with different excitation frequencies. From the simulations, the response of the sensor probe was more evident to see the changes of the magnetic B field using higher excitation frequency. Besides, since the maximum depth of the crack was 2 mm, in order to have a better performance of crack detection and full penetration of the sample plate (the skin depth of the eddy current at 20 kHz was slightly larger than 2 mm), therefore, the frequency was chosen to be 20 kHz.

**Figure 4:**
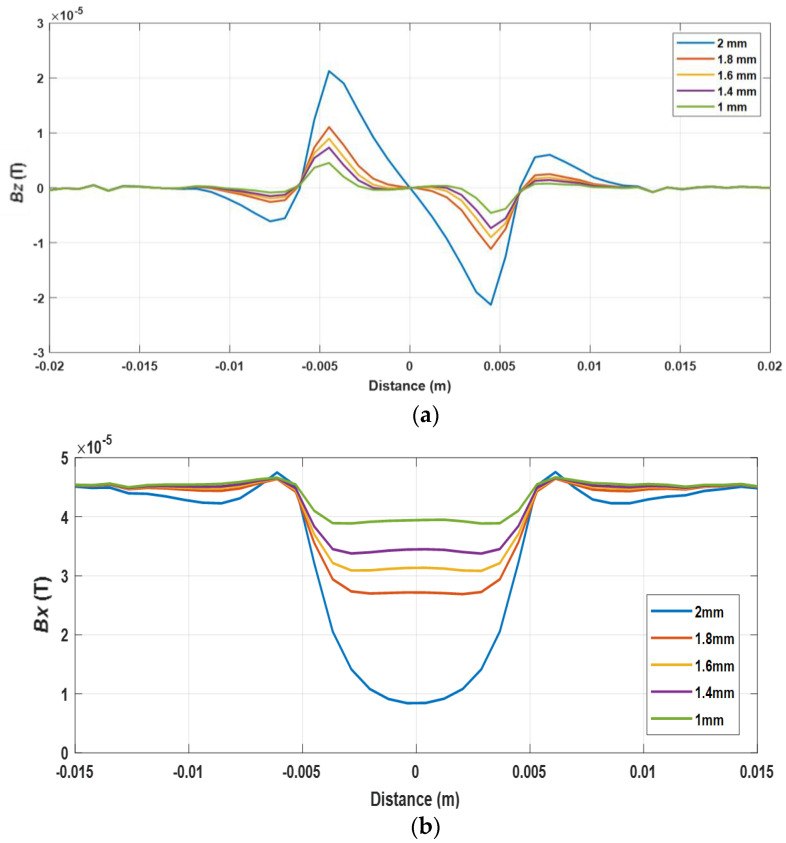
The magnetic B field signal received by the sensing probe under different depths of the cracks (**a**) $B_{z}$, (**b**) $B_{x}$.

Figure 4 illustrates the x and z components of the magnetic B field caused by the cracks with different depths varying from 1 to 2 mm under the excitation frequency of 20 kHz. It can be seen that, in the center of the crack, the z component of the magnetic B field was zero while the x component of the magnetic B field reached the minimum value. It can be observed that, the deeper the crack, the larger the peak value of $B_{z}$ and $B_{x}$.

**Figure 5:**
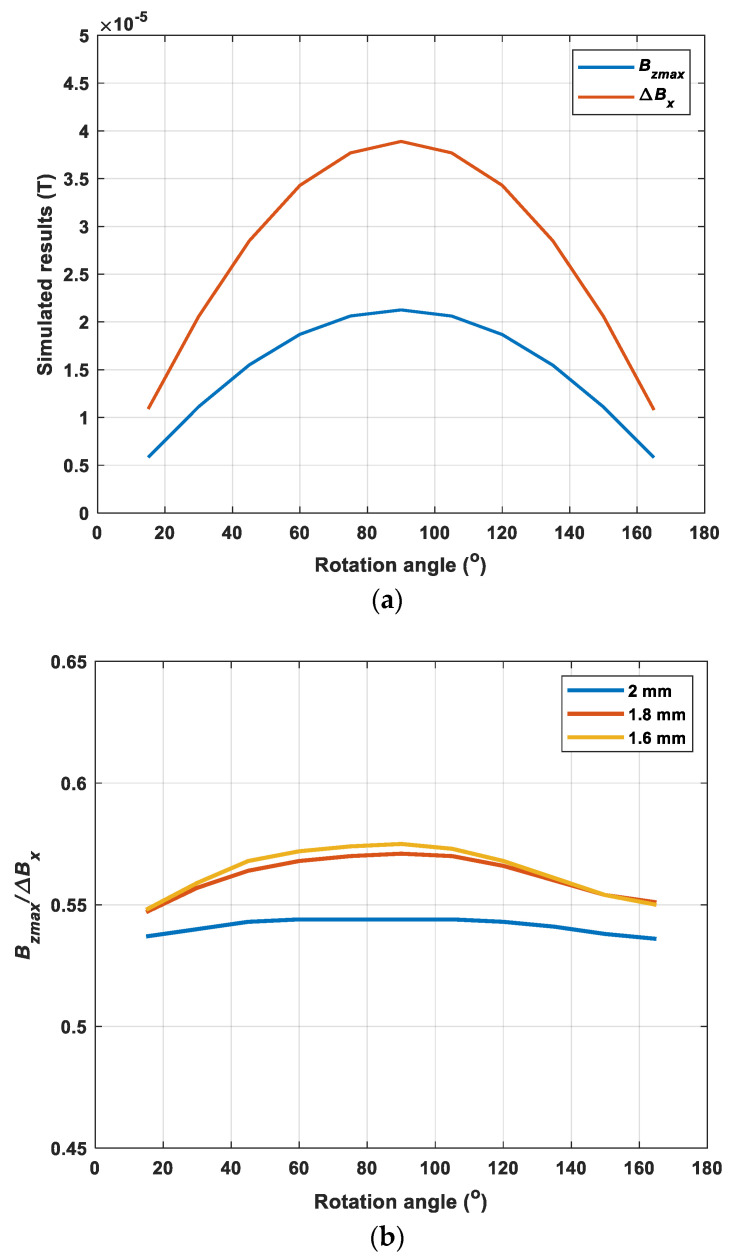
(**a**) The simulated results of the magnetic B field for the crack depth of 2 mm. (**b**) The ratio of the maximum of the z component and the change of the x component under varying rotation angles for different crack depths.

**Figure 6:**
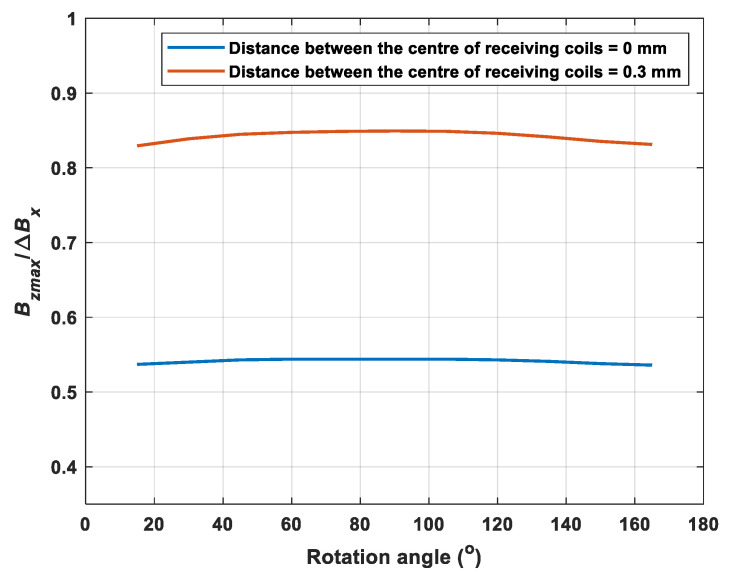
The ratio of the maximum of the z component and the change of the x component under varying rotation angles for different distance between the centre of receiving coils with the crack depth of 2 mm.

Further, the sensor probe scanned across the crack with a range of rotation angles starting from 15 degrees (one period) in steps of 15 degrees to investigate the variation of the two components of the magnetic field. Figure 5a depicts the variations of the maximum value of ***B_z_*** and the difference of $B_{x}$ with the changing rotation angle. It can be noticed that the trend of the peak value of $B_{z}$ and the difference of $B_{x}$ was similar. Both of them increased at the beginning, then reached their maximum at the angle of 90° and decreased again. They followed a sine relationship between the rotation angle and the x/z component of the magnetic B field. As can be seen from Figure 5b, the ratio of the maximum of the z component ($B_{z}$) and the change of the x component ($B_{x}$) stayed constantly under a range of angles. It can be noted that the ratio was nearly immune to the rotation angle with reasonable variation (3%) and the value increases under different depths of the crack. Besides, when the center of receiving coils was not overlapped (i.e., the receiving coil for detecting x component of the magnetic B field was placed with higher lift-off, above the receiving coil for detecting z component of the magnetic B field), the ratio increased from 0.54 to 0.83, as shown in Figure 6. The reason for the increase of the ratio was because, with a higher lift-off of the receiving coil for detecting x component of the magnetic B field, the difference of $B_{x}$ became smaller so that the ratio increased but still remained stable under varying rotation angles. It showed the potential of using this ratio to determine the depth of the crack by reducing the effect of the crack orientation.

## 3. Experiments

### 3.1. Experimental Setup

**Figure 7:**
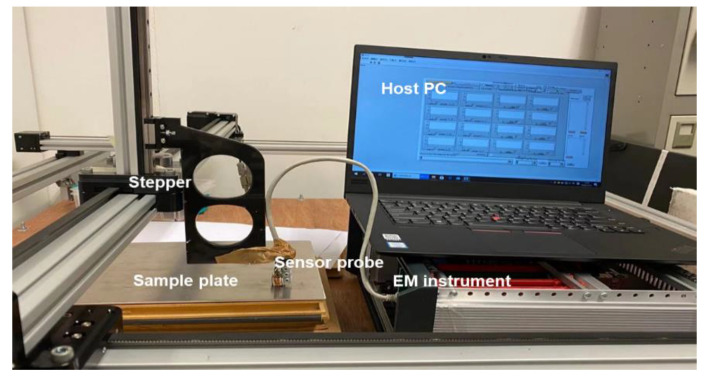
Experimental setup.

Figure 7 shows the experimental setup for crack detection, consisting of a stepper, the electromagnetic (EM) instrument, host PC, sensor probe, and the sample plate. The sensor probe was attached to the stepper, whose movements in different axis can be controlled by the host PC to perform the scanning process. The sample plate was fixed at the stage and the sensor probe was right on the top of the cracks during scanning. The experiment parameters are listed in Table 1. The magnetic field was detected by the EM instrument developed by the Sensing, Imaging and Signal Processing group at the University of Manchester [27,28].

**Figure 8:**
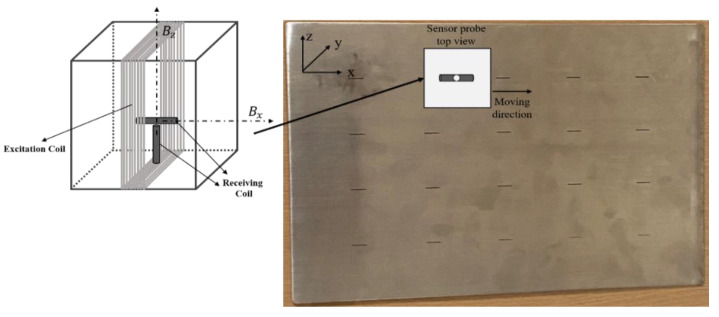
The trajectory of the sensor probe.

The EM instrument is based on field programmable gate array (FPGA) programming which enables its high speed for the detecting process. As shown in Figure 1a and Table 2, the excitation coil was vertically placed above the sample plate (lift-off of 1.5 mm) with 20 turns. The length and height of the excitation coil were 12 and 10 mm, respectively. As is shown in Figure 1 and Figure 8, two receiving coils are assembled in the center of the excitation coil along the X-axis and the Z-axis, respectively, for detecting the z and x components of the magnetic B field. The radius and turns of the receiving coil were 0.8 mm and 200, respectively. The stainless-steel sample plate was used which contained 20 small cracks with a length of 10 mm and a width of 0.25 mm. The depth of the crack is from 0.1 to 2 mm, with an increasing step of 0.1 mm.

**Figure 9:**
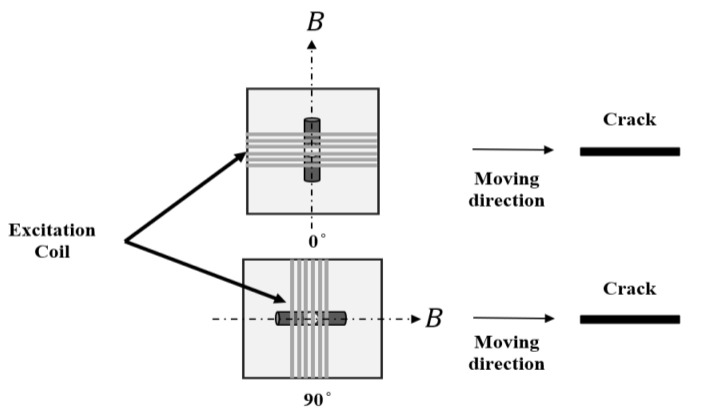
The orientation of the sensor probe at 0° and 90°.

During the scanning process, the sensor probe moved right on the top of the crack along the X-axis, which performed line scanning. The trajectory of the sensor probe covered the total length of the crack. In Figure 8, the angle between the excitation coil and the X-axis was 90° and a maximum $B_{x}$ can be detected. During the experiments, the sensor probe was rotated, and line scanning was performed at different angles. In Figure 9, the orientation of the sensor probe at 0° and 90° is demonstrated, respectively. The direction of the induced magnetic field varies with the orientation of the excitation coil, which results in different levels of change in the measurements at each angle.

**Figure 10:**
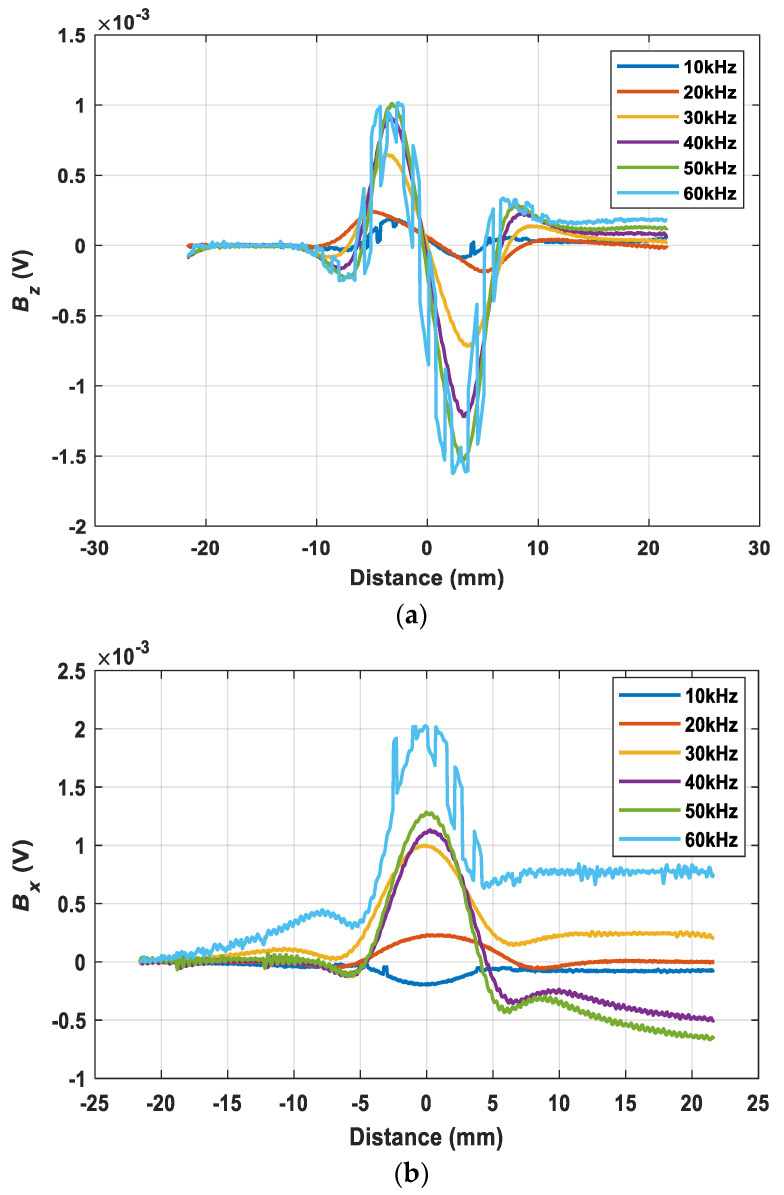
The voltage received from the sensor probe under different frequencies (**a**) $B_{z}$, (**b**) $B_{x}$.

**Figure 11:**
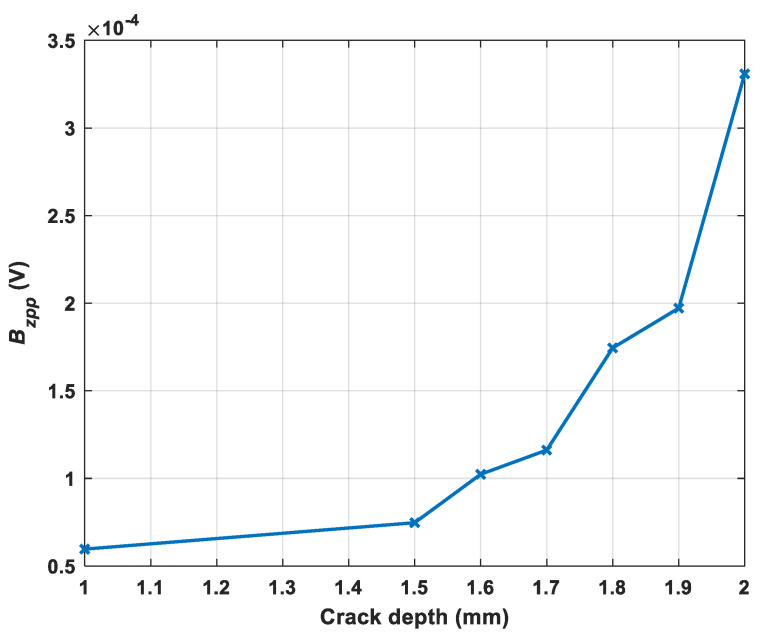
The peak-to-peak value of z component of the magnetic field with different crack depths under the frequency of 20 kHz.

The measured results were presented by the difference of the received voltage of the sample with the crack and without the crack. As shown in Figure 10, the excitation frequency from 10 to 60 kHz is used for testing the crack with a depth of 2 mm. It can be noted that frequency ranges from 20 to 50 kHz works well and have a better SNR. Therefore, 20 kHz was selected as the excitation frequency. Besides, the excitation frequency of 20 kHz works for different depths of crack is shown in Figure 11 and it can be noticed that there was a rising trend of the peak-to-peak value of the z component of the magnetic field as the crack depth increases.

### 3.2. Coil-Crack Angle Insensitive Feature

**Figure 12:**
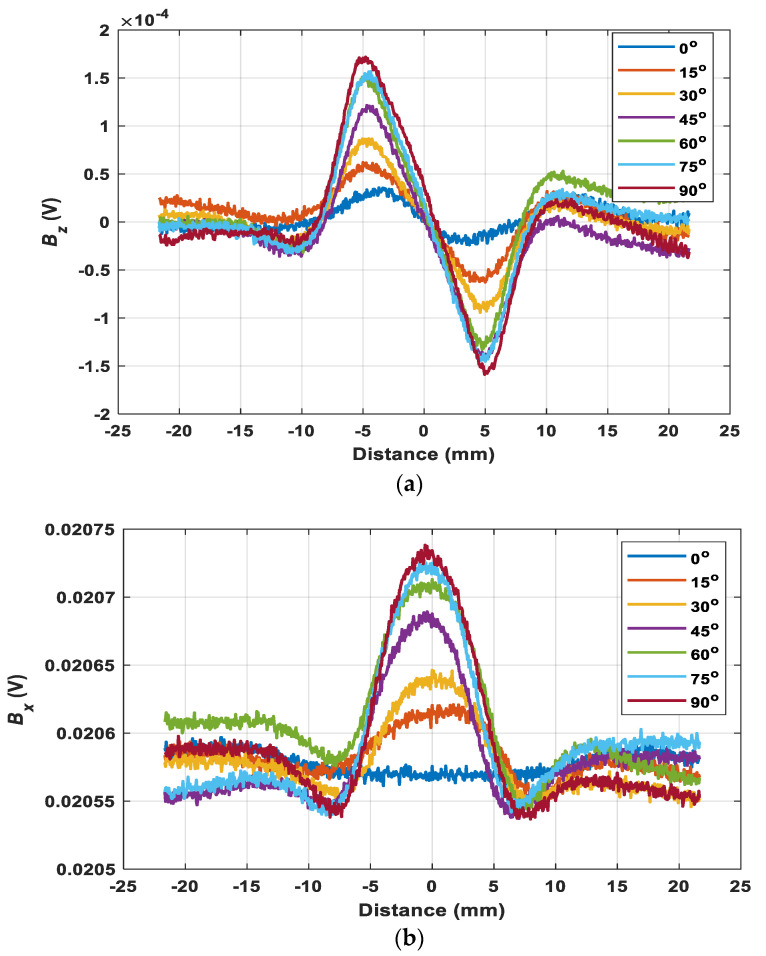
The measurement results of the magnetic field under the rotation angle from 0° to 180° (**a**) the z component, (**b**) the x component.

**Figure 13:**
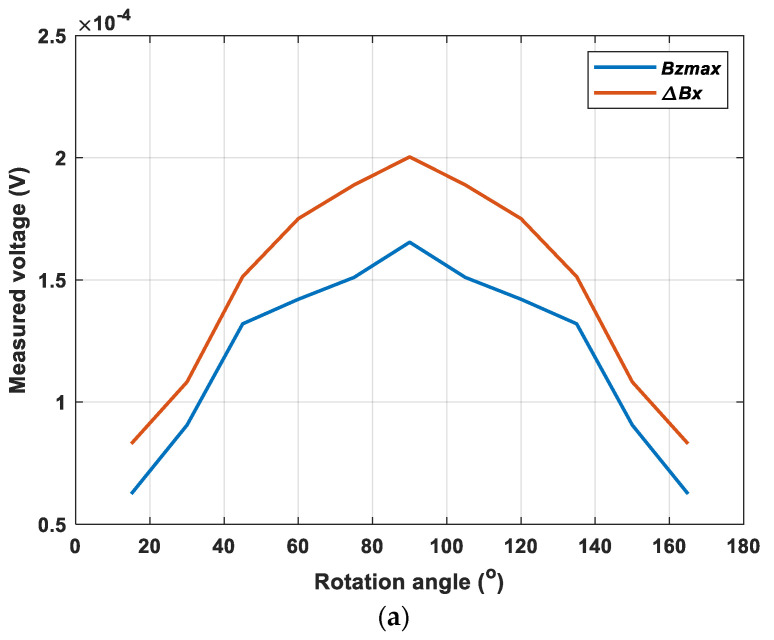

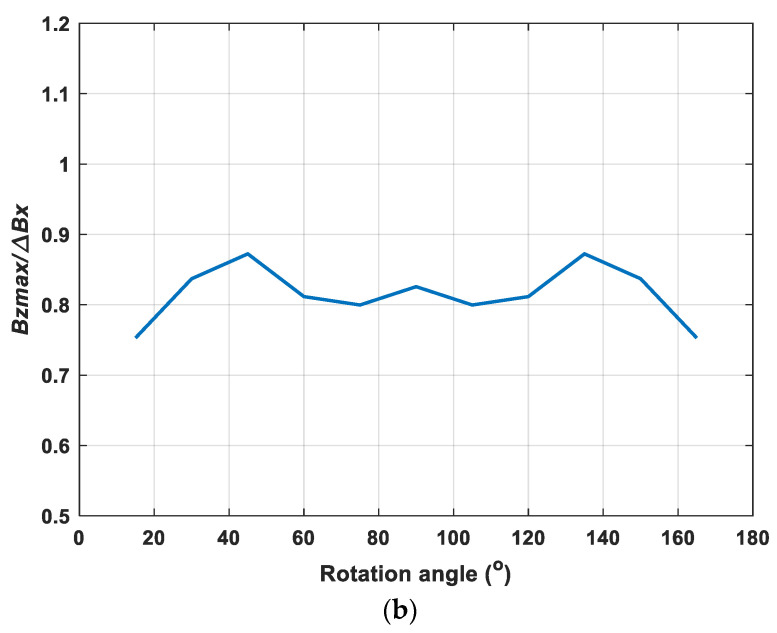
(**a**) The measured results of the magnetic B field. (**b**) The ratio of the maximum of the z component and the change of the x component under varying rotation angle.

As can be seen in Figure 12, the trend of the magnetic field under different rotation angles is similar compared with the simulation results. Figure 13 shows the received maximum value of the z component and the change of x component of the magnetic B field with a range of rotation angles. The value of the x and z components of the magnetic B field increased with the rotation angle from 15°, reaching maximum when the rotation angle is 90°. This is because more eddy current was blocked (the flowing route is influenced) due to the presence of the crack as the sensor probe rotates vertically to the crack. Then continuing to rotate the sensor probe, the value reversely decreased to reach its minimum. They agreed with the simulated results which were symmetric with respect to 90°. Moreover, it can be seen in Figure 13b that there was a coil angle immune feature for the ratio of the maximum of $B_{z}$ and the change of $B_{x}$ when the sensor probe scans the crack on the surface of the metal. Due to the measurement error and the environments, there was a small fluctuation for the ratio, but most of them were mainly around 0.8, which was consistent with the simulated results. Besides, it also showed that the position of the receiving coil for detecting x component of the magnetic B field did not influence the coil-crack angle insensitive feature. Therefore, the effect caused by the coil-crack angle can be eliminated by the feature and may be useful to estimate the depth of the crack without the disturbance of the crack orientation.

## 4. Conclusions

In this paper, a novel crack detection method by using rotated ACFM techniques was proposed. The proposed method was using the sensor probe with two orthogonal receiving coils to detect the magnetic B field under different rotation angles. It was found that there was a sine relationship between the peak value of the z component/the change of the x component of the magnetic B field and the rotated angle. Besides, it is noted that the ratio of the peak value of the z component and the change of x component stayed constantly under different rotation angles. It was also validated by the measurement results. By utilizing this feature, it may be used to determine the depth of the crack reducing the effect of the crack orientation for the conductive metallic plate. Moreover, with the support of the proposed method, it simplified the sensor setup at low cost.

## Figures and Tables

**Table 1:** Model parameters.

	Values
Height of excitation coil h (mm)	3
Length of excitation coil l (mm)	4
Turns of excitation coil $N_{e}$	5
Radius of receiving coils $r_{p}$ (mm)	0.5
Turns of receiving coils $N_{p}$	1
Lift-off $l_{o}$ (mm)	0.5
Width of the sample plate $w_{s}$ (mm)	75
Length of the sample plate $l_{s}$ (mm)	40
Thickness of the sample plate c (mm)	2
Width of the crack $w_{c}$ (mm)	10
Length of the crack $l_{c}$ (mm)	0.25
Conductivity of the sample plate σ (MS/m)	1.4

**Table 2:** Experimental parameters.

Excitation coil	Length (mm)	12
	Height (mm)	10
	Turns	20
Receiving coil	Radius (mm)	0.8
	Turns	200
Lift-off (mm)		1.5
Excitation frequency (kHz)		20
Crack depth (mm)		0.1:0.1:2

## Data Availability

Not applicable.
